# Optic nerve head astrocytes contribute to vascular associated effects

**DOI:** 10.3389/fmed.2022.943986

**Published:** 2022-07-26

**Authors:** Yanmin Dong, Yue Fu, Xiaobing Qian, Leilei Lin, Yongguang Yuan, Yujie Li, Wanwen Shao, Qianying Gao

**Affiliations:** ^1^Department of Ophthalmology, The First Affiliated Hospital of Zhengzhou University, Zhengzhou, China; ^2^State Key Laboratory of Ophthalmology, Zhongshan Ophthalmic Center, Sun Yat-sen University, Guangzhou, China

**Keywords:** astrocytes, optic nerve head, vascular regulation, endothelin-1, nitric oxide synthase, renin-angiotensin system

## Abstract

**Purpose:**

This study was conducted in order to test the expression of vasoactive substances within rat lamina cribrosa (LC) and optic nerve head (ONH) astrocytes, so as to investigate the role and potential mechanism of ONH astrocytes in vascular associated effects.

**Methods:**

LC tissue sections and primary cultured ONH astrocytes were obtained from adult Sprague-Dawley (SD) rats. Immunofluorescent staining was then used to detect the expression of vasoactive substances. Hyperoxia exposure was carried out both *in vivo* and *in vitro*, after which nitric oxide (NO) levels in LC tissue and cell supernatant were detected. The variations of protein and gene expression associated with vasoactive substances were subsequently tested. ONH astrocytes and vascular smooth muscle cells (VSMCs) were then incubated in a direct co-culture manner. Morphological parameters of VSMCs were finally analyzed in order to evaluate cell contraction.

**Results:**

Endothelin-1 (ET-1), nitric oxide synthase (NOS) and renin-angiotensin system (RAS) were detected in both LC tissue and ONH astrocytes. Retinal vessel diameter was found obviously decreased following hyperoxia exposure. Moreover, hyperoxia inhibited NO production both *in vivo* and *in vitro*. ET-1 and RAS elements were observed to be upregulated, whereas NOS was downregulated. In ONH astrocytes and VSMCs co-culture system, the length-to-width ratio of VSMCs was shown to significantly increase on days 3 and 7 in hyperoxia compared with normoxia.

**Conclusions:**

There is an abundance of expression of vasoactive substances within LC tissue and ONH astrocytes. The contractile response of VSMCs in the co-culture system provided direct evidence for the involvement of ONH astrocytes in vascular associated effects, which may signify a potentially novel direction for future research.

## Introduction

The lamina cribrosa (LC) of the optic nerve head (ONH) serve as the definite site in which the central retinal artery and vein (CRA and CRV) pass through to enter or leave the eye. The LC region is composed of glial columns and connective tissue plates. Two distinct cell types are believed to serve the LC: ONH astrocytes and LC cells (1, 2). Studies have also suggested the presence of paracrine and/or autocrine signaling within the LC(3–5).

Glial cells have been shown to contribute to basal retinal vascular tone and modify the dynamic response of arteries and veins toward changes in perfusion pressure (6). Recent studies have found that the perfusion pressure of ONH decreased significantly when intraocular pressure was artificially elevated in gliotoxic compound treated rabbits, whereas blood flow was maintained in control eyes. These findings indicated the possible involvement of glial cells in the autoregulation of ONH blood flow (7). Astrocytes, in addition to these supportive roles, may play a central role in many other aspects of brain function, including neuronal signal regulation and cerebrovascular regulation (8, 9). The optic nerve is considered to be a continuation of the central nervous system. Accordingly, certain structural similarities may indicate that ONH astrocytes play a similar role in the eye.

Retinal arteries differ from arteries of the same size in other organs as they have an unusually developed smooth muscle layer and lack an internal elastic lamina. The arterial wall near the optic disk is composed of five to seven layers of smooth muscle cells. Additionally, the presence and distribution of retinal astrocytes have been shown to possess high consistency with the presence and distribution of retinal blood vessels, while only vascularized retina contain astrocytes (10). Astrocytes in the diffusely vascularized retina have been posited to be derived from the optic nerve (11). Morover, astrocytes have an intimate structural relationship with the retinal microvasculature. The endfoot processes of astrocytes completely envelop all retinal blood vessels (12). In this regard, such natomical structure and spatial distribution characteristics in ONH astrocytes may provide certain advantages in vascular regulation.

Astrocytes, which link neurons to blood and provide structural and physiological support to optic nerve head axons, are essential for the maintenance of ocular tissue homeostasis (13, 14). Therefore, this study aims to determine whether ONH astrocytes express vasoactive factors and investigate the role and potential mechanism of ONH astrocytes in vascular associated effects. Such knowledge would help explore the pathogenesis of neurodegenerative diseases such as glaucoma from a new perspective.

## Materials and methods

### *In vivo* study

#### Experimental animals

Sprague-Dawley (SD) rats were obtained from the institutional animal facility and were raised in dim cyclic illumination (12 h: 12 h light-dark cycle) at 24°C. All animal experiments were performed in accordance with the Association for Research in Vision and Ophthalmology Statement for the Use of Animals in Ophthalmic and Vision Research.

#### Architecture and histopathological analysis of healthy rat LC

Eyeballs were enucleated and fixed in 4% paraformaldehyde (PFA) for 48 h (anterior segment was removed at 2 h), dehydrated with sucrose, and then embedded in OCT for frozen section. Sections through the ONH and retrobulbar optic nerve were cut with a thickness of 5 μm on a cryo-ultramicrotome (CM1850; Leica, Germany). Both horizontal and vertical longitudinal sections were cut through the ONH for architecture and histopathological analysis. All cryosections were collected on polylysine coated glass slides and stored at −80°C until HE staining and immunofluorescence analysis.

#### Immunofluorescent detection of vasoactive substances in rat LC tissue

Frozen sections of rat LC were first fixed in 4% PFA for 15 min at room temperature, solubilized with phosphate-buffered saline (PBS) with 0.1% Triton X-100 (PBST). They were then blocked in 5% bovine serum albumin (BSA) for 60 min at 37°C and incubated with primary antibodies (Table 1) or PBS (negative controls) at 4°C overnight. Next, sections were washed with PBS, and incubated in appropriately labeled (Alexa Fluor® 488, Alexa Fluor® 647) secondary antibodies at 37°C for 45 min. After three washes in PBS, sections were treated with DAPI nuclear stain at room temperature for 5 min, washed, and mounted.

**Table 1:** List of antibodies.

**Antibody**	**Dilution and application**	**Source**	**Cat #**
Rabbit polyclonal anti-glial fibrillar acid protein (GFAP)	• 1/1,000 IHC • $\frac{1}{4}$,000 ICC	Abcam	ab7260
Rabbit polyclonal anti-laminin (LN)	• 1/100 IHC • 1/100 ICC	Abcam	ab7463
Mouse monoclonal anti-endothelin 1(ET-1)	• 1/200 IHC • 1/200 ICC • 1/500 WB	Abcam	ab2786
Mouse monoclonal anti-nitric oxide synthase 1 (NOS1)	• 1/50 IHC • 1/50 ICC • 1/200 WB	Santa Cruz	sc-5302
Rabbit polyclonal anti-nitric oxide synthase 2 (NOS2)	• 1/50 IHC • 1/50 ICC	Santa Cruz	sc-651
Mouse monoclonal anti-nitric oxide synthase 3 (NOS3)	• 1/50 IHC • 1/50 ICC • 1/100 WB	Santa Cruz	sc-376751
rabbit polyclonal anti- guanylate cyclase(GC)	• 1/50 IHC • 1/300 WB	Abcam	ab53084
RABBIT polyclonal anti-renin	• 1/200 IHC • 1/200 ICC • 1/1,000 WB	Abcam	ab137731
Rabbit polyclonal anti-angiotensin (Ang)	• 1/200 IHC • 1/200 ICC	Abcam	ab89892
Rabbit monoclonal anti-angiotensinogen (AGT)	• 1/100 IHC • 1/100 ICC • 1/1,000 WB	Abcam	ab108334
Mouse monoclonal anti-angiotensin converting enzyme 1 (ACE1)	• 1/100 ICC	Abcam	ab77990

*ICC, immunocytochemistry; IHC, immunohistochemistry; WB, western blot*.

#### Exposure of adult rats to consecutive hyperoxia

Healthy SD rats aged 8 weeks were used in this procedure, which were randomly divided into two groups: the hyperoxia and normoxia groups. All rats were placed in a clear plexi glass chamber and were exposed to consecutive hyperoxia (75% O_2_) and normoxia (room air, 21% O_2_) for up to 7 days. Oxygen levels were maintained using an Oxycycler feedback-controlled device (Biospherix, Lacona, NY).

#### Retinal vessel diameter measurement

Fundus imaging and fundus fluorescein angiography (FFA) were then performed in order to evaluate the retinal vessel diameter before and after hyperoxia exposure. The SD rats were routinely anesthetized and the pupils were dilated. After intraperitoneal injection of fluorescein sodium (10%, 0.15 ml/100 g), dynamic fundus photographs were taken using a retinal imaging microscope (Phoenix Micron IV, Phoenix, USA). The diameter of retinal vessels beside the ONH edge, and at one time the optic disc diameter, was measured using Image J. One eye of each rat was randomly selected for statistical analysis. The same vessel segment was measured three times to take the average.

#### Detection of nitric oxide level in tissues

In each group, five rats were euthanized at the endpoint, and both eyes were enucleated. The retina and LC tissue were then isolated under an ophthalmic anatomic microscope and were homogenized in sodium dodecyl sulfate (SDS) lysis buffer (P0013G; Beyotime, Beijing, China). The centrifugal supernatant of each sample was used for nitric oxide (NO) level detection using NO detection kit (S0021, Beyotime, Beijing, China) according to the manufacturer's instructions. Optical density was measured at 540 nm by a Multi-Mode Microplate Reader (Synergy H1, BioTek, USA). The level of NO was expressed as μmol/L.

#### Preparation of protein and western blot analysis

At the endpoint, LC and retina tissue protein were extracted in lysis buffer (5 μL/mg) (P0013G; Beyotime, Beijing, China) according to the manufacturer's instructions. The homogenates were then completely lysed on ice for 30 min and centrifuged at 13,000 rpm at 4°C for 20 min. The supernatants were collected, and the protein concentration was tested using a BCA protein assay kit (23212; Pierce, USA). Fifteen microns of protein samples were loaded and separated on gradient gels, which were then transferred onto a polyvinylidene difluoride (PVDF) membrane *via* electrophoresis. PVDF membranes were subsequently blocked with 5% BSA for 3 h at room temperature, and then incubated with specific primary antibodies (Table 1) at 4°C overnight and corresponding horseradish peroxidase (HRP)-conjugated secondary antibodies for 2 h at room temperature. Immunoreactive bands were detected using a diaminobenzidine detection kit (Boster Biotechnology, Wuhan, China). The intensity of the stained bands was quantified by Image J. Each test was repeated three times and the mean intensity was used for the statistical analysis.

#### Isolation of total RNA and real-time PCR

Total RNA was prepared from the retina and LC of rat using TRIzol (Invitrogen, Carlsbad, CA) according to the manufacturer's instructions. Quantification of total RNA was performed using the NanoDrop 2000 (Thermo Scientific, Wilmington, DE). An absorbance ratio of RNA at 260 and 280 nm was then used to guide the determination of RNA purity. Total RNA with a ratio of 1.8 or more was considered to be acceptable, which was reverse transcribed to generate cDNA. Quantitative PCR was performed (Applied Biosystems 7500; Applied Biosystems CO., U.S.A.) with Power SYBR Green (RR420A, SYBR® Premix Ex Taq^TM^, Takara, Dalian, China). The β-actin gene was used as an internal reference. The expressions of purpose genes were quantified using 2^−Δ*ΔCt*^, and data was collected from three independent experiments. Primer sequences for rat transcripts are shown in Table 2.

**Table 2:** Primers used for real-time PCR.

**Gene name**	**Forward primer (5**′**–**>**3**′**)**	**Reverse primer (5**′**–**>**3**′**)**	**TM**°**C**	**Amplicon size**
β-actin	GAGAGGGAAATCGTGCGT	GGAGGAAGAGGATGCGG	60	93
ET-1	ACTCCGAGCCCAAAGTACC	TAGTTTTCTTCCCTCCACCAG	60	115
GC	AGTGTCCTTTCTCCTTACTGGC	GTAGACTCTGTTGCGGCTTGT	60	167
NOS1	AATCACAAGCCTATGCCAAGAC	CCATTAAAGCACAGCCGAAT	60	186
NOS2	CAGCATCCACGCCAAGAA	CAACTCGCTCCAAGATCCCT	60	200
NOS3	CAGCATCCACGCCAAGAA	CAACTCGCTCCAAGATCCCT	60	152
Renin	CAAGTTTGACGGGGTTCTGG	CAAGTTTGACGGGGTTCTGG	60	199

### *In vitro* study

#### Primary culture of ONH astrocytes

Rat LC explants were obtained and cultured as described by previous studies (2, 4, 15). Primary cell extraction was performed from 20 eyes of 10 rats. Two-month-old male SD rats were euthanized, and the ONH was carefully dissected and washed in a 35 mm dish containing ice-cold 0.1 M PBS pH 7.4 (PBS; Lonza, Walkersville, MD) 5 × strength penicillin/streptomycin (P/S) (500 U/mL of penicillin, 500 g/mL streptomycin; Gibco, Invitrogen Corp, Grand Island, NY). The LC is located in the middle of the optic disc, with a translucent constricted area appearance. Nerve fibers at post laminar region are myelinated and increase in thickness; therefore, the boundary between myelinated and unmyelinated region can be used as an anatomical marker. The LC tissue was dissected from the pre- and retro-laminar region under a dissecting microscope and was cut into three to four explants and plated in 12-well plate containing 2 ml Dulbecco modified eagle medium (DMEM, Gibco, Invitrogen Corp) supplemented with 20% fetal bovine serum (FBS, Gibco, Invitrogen Corp) and 1 × strength P/S. Cells that grew out of the LC explants contain ONH astrocytes and LC cells. The two cell types were then separated by the serum-free culture method. Cells expressing glial fibrillar acid protein (GFAP) and lamin (LN) were characterized as ONH astrocytes (4, 15). The purified ONH astrocytes were maintained in astrocyte growth medium (AGM) containing 5% FBS and were passaged. Cells between passages four and six were then used for the subsequent experiments (16).

#### Primary culture of vascular smooth muscle cells

VSMCs in primary culture were obtained from the aortic media of SD rats weighing 150–180 g (17). The cell phenotype was evaluated by morphology and immunofluorescence of smooth muscle myofilament proteins: rabbit polyclonal anti-alpha-smooth muscle actin (α-SMA) (ab5694, 1/200, Abcam). The experiments were then performed on cells between passages four and six.

#### Immunofluorescent detection of vasoactive substances in rat ONH astrocytes

Plated ONH astrocytes were rinsed with PBS, fixed with 4% PFA for 15 min, and solubilized with 0.1% Triton X-100 for 15 min.

The subsequent experimental steps were performed according to the vivo experiment section.

#### Exposure of ONH astrocytes to consecutive hyperoxia

Confluent ONH astrocytes were maintained in serum-free media under conditions of hyperoxia (75% O_2_, 5% CO_2_, 37°C) and normoxia (21% O_2_, 5% CO_2_, 37°C). The cell culture chamber of hyperoxia was atmospherically sealed, and the inward gas was strictly regulated by an oxygen and carbon dioxide controller (C21, BioSpherix, Ltd, Lacona, NY).

#### RNA preparation and real-time PCR

Gene expression of ONH astrocytes in normoxia and hyperoxia condition was detected by real-time PCR. The details have been introduced in the front.

#### NO level assay

After ONH astrocytes were treated with 75%O_2_ for 1, 3, and 7 d, the NO level in supernatant of the co-culture system was tested using the NO detection kit according to the manufacturer's instructions which has been described in the front part.

#### Direct co-culturing of ONH astrocytes and VSMCs

In the direct co-culture system, ONH astrocytes were grown to confluence on the bottom of 60 mm culture dishes. VSMCs were grown on glass coverslips that were placed on a 24-well plate, at a density of 100,000 cells per well. At confluence, the glass coverslips with VSMCs were placed on the confluent monolayer of the ONH astrocytes. The medium was replaced with DMEM (10%FBS, 1%Ab). All plates were then incubated for 24 h in a humidified incubator under normal cell culture conditions of 37°C, 5% CO_2_ and 21% O_2_ (95% air). Then, the separate co-cultures plates were transferred to either a humidified culture chamber maintained at 37°C, 5% CO_2_ and 75% O_2_ or an incubator maintained under normal cell culture conditions of 37°C, 5% CO_2_ and 21% O_2_. The individual VSMCs continued to grow as the control.

#### Morphometric analysis of VSMCs

VSMCs in hyperoxia and normoxia at each time point were disposed with Immunofluorescent Staining. The α-SMA Positive Staining Cells Were analyzed using the Image pro plus software (v6.0, Media Cybernetics, Bethesda, MD). Morphological parameters contained cell area, cell length and cell width, and the length-to-width ratio was estimated. The mean values were derived from at least 5 cells per high magnification field and at least 10 fields per group.

#### Statistical analysis

A statistical analysis was performed using the SPSS Statistical Software, version 25.0 (SPSS Inc. Chicago, USA). *T*-test and ANOVA were applied to test for differences between groups. The statistical analysis was also conducted in GraphPad Prism for Windows, version 6.00 (GraphPad Software Inc., La Jolla, Calif., USA). Data were expressed as mean ± standard deviation (SD). For all analyses, *P* < 0.05 was considered to be statistically significant.

## Results

### *In vivo* results

#### Expression of vasoactive substances within LC tissue

**Figure 1:**
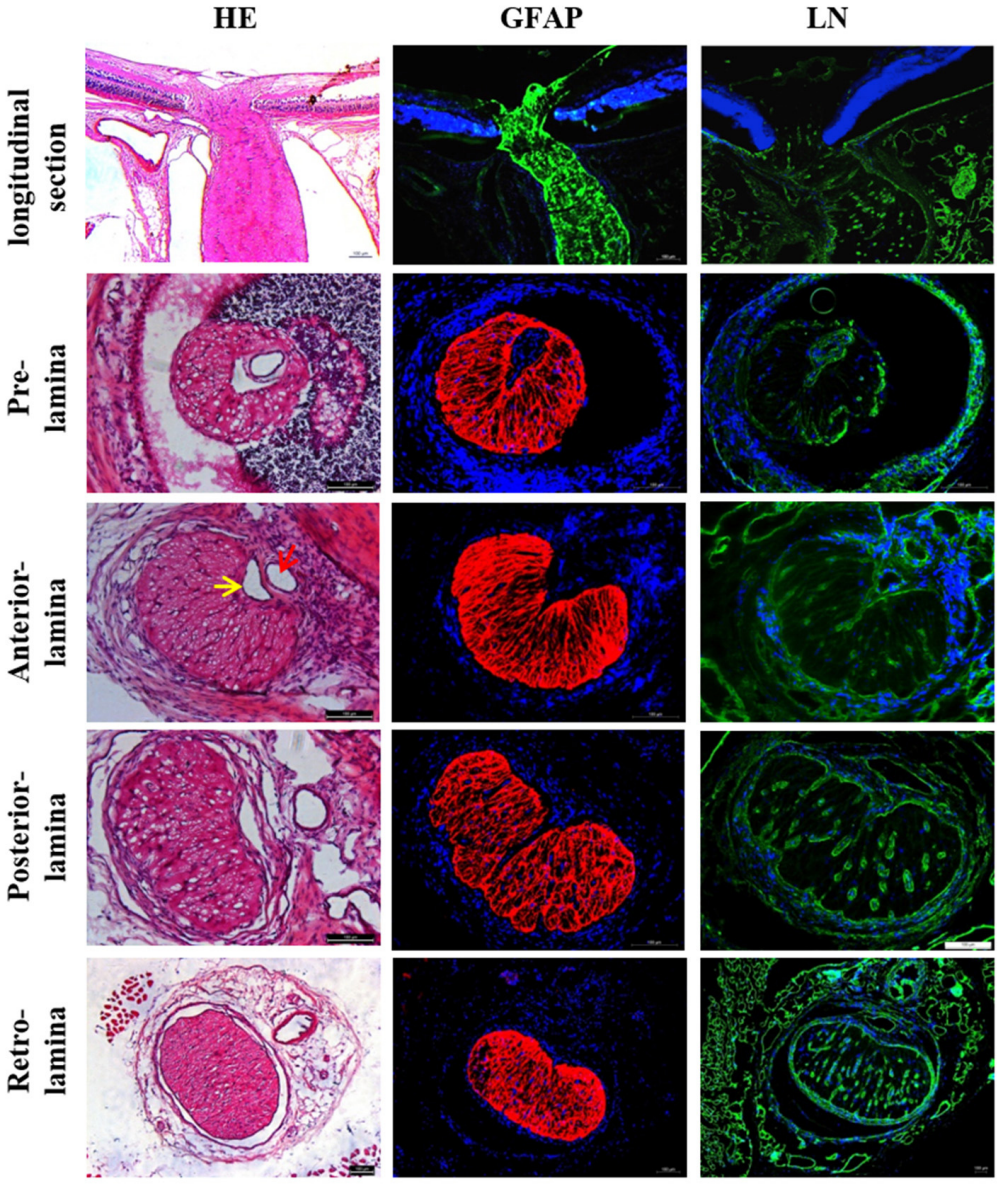
Histological characteristics of rat LC. LC was located in the front of the optic nerve, which narrowed like a “bottle neck.” Rat LC exhibited a well-developed lamellar structure and positive staining against GFAP and LN. CRA (red arrow) and CRV (yellow arrow) are closely adjacent in the laminar region. The lumen of CRA was nearly round, whereas the lumen of CRV had a triangular or crescentiform configuration. LC, Lamina cribrosa; GFAP, Glial fibrillar acid protein; LN, Laminin; CRA, Central retinal artery and vein; CRV, Central retinal vein.

**Figure 2:**
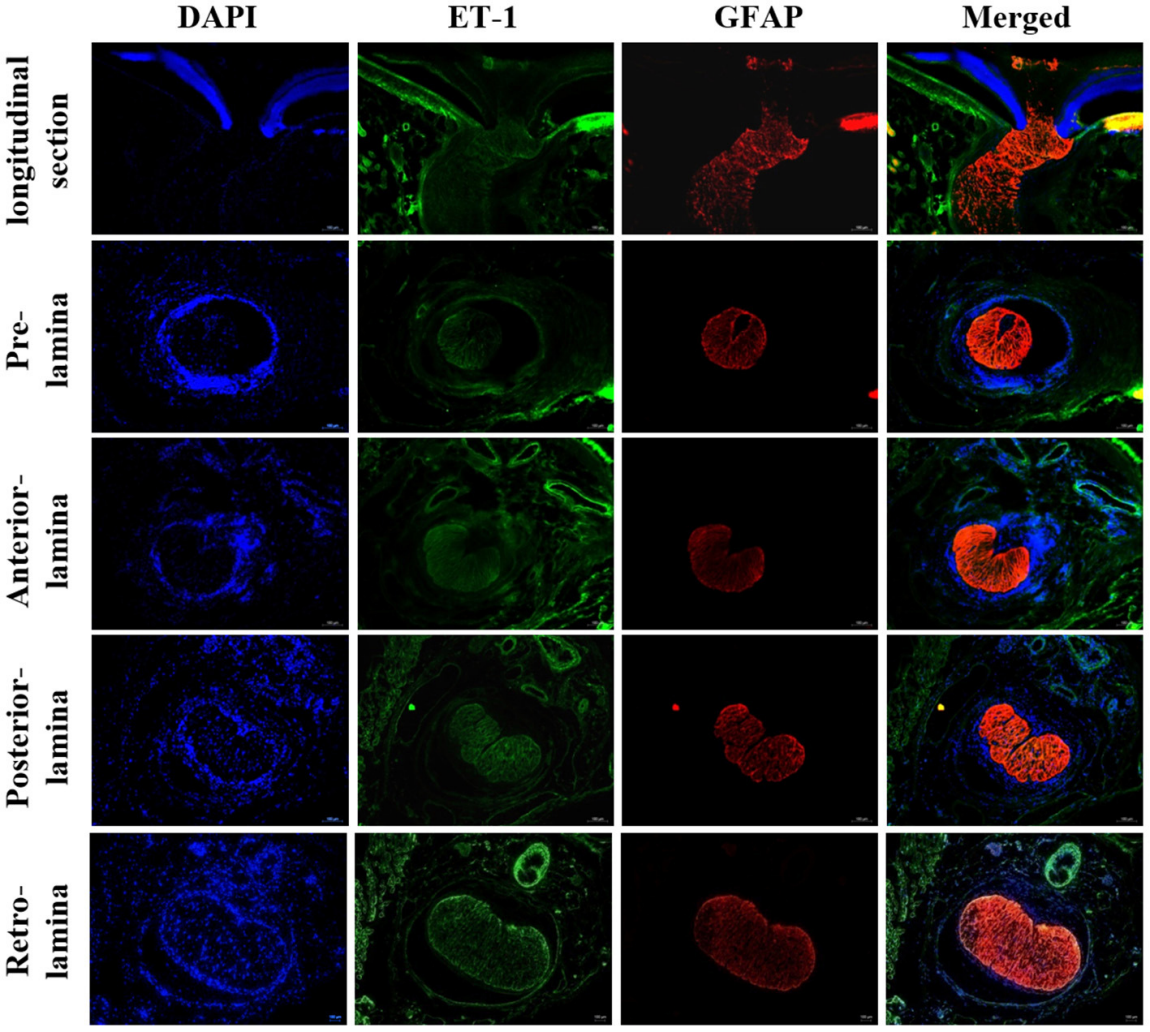
Immunofluorescent staining for ET-1 in rat LC tissues. Staining for ET-1 was mainly located in the surface layer of ONH, LC, retrobulbar optic nerve, and central retinal vessels. ONH, Optic nerve head; LC, Lamina cribrosa; ET-1, Endothelin 1.

**Figure 3:**
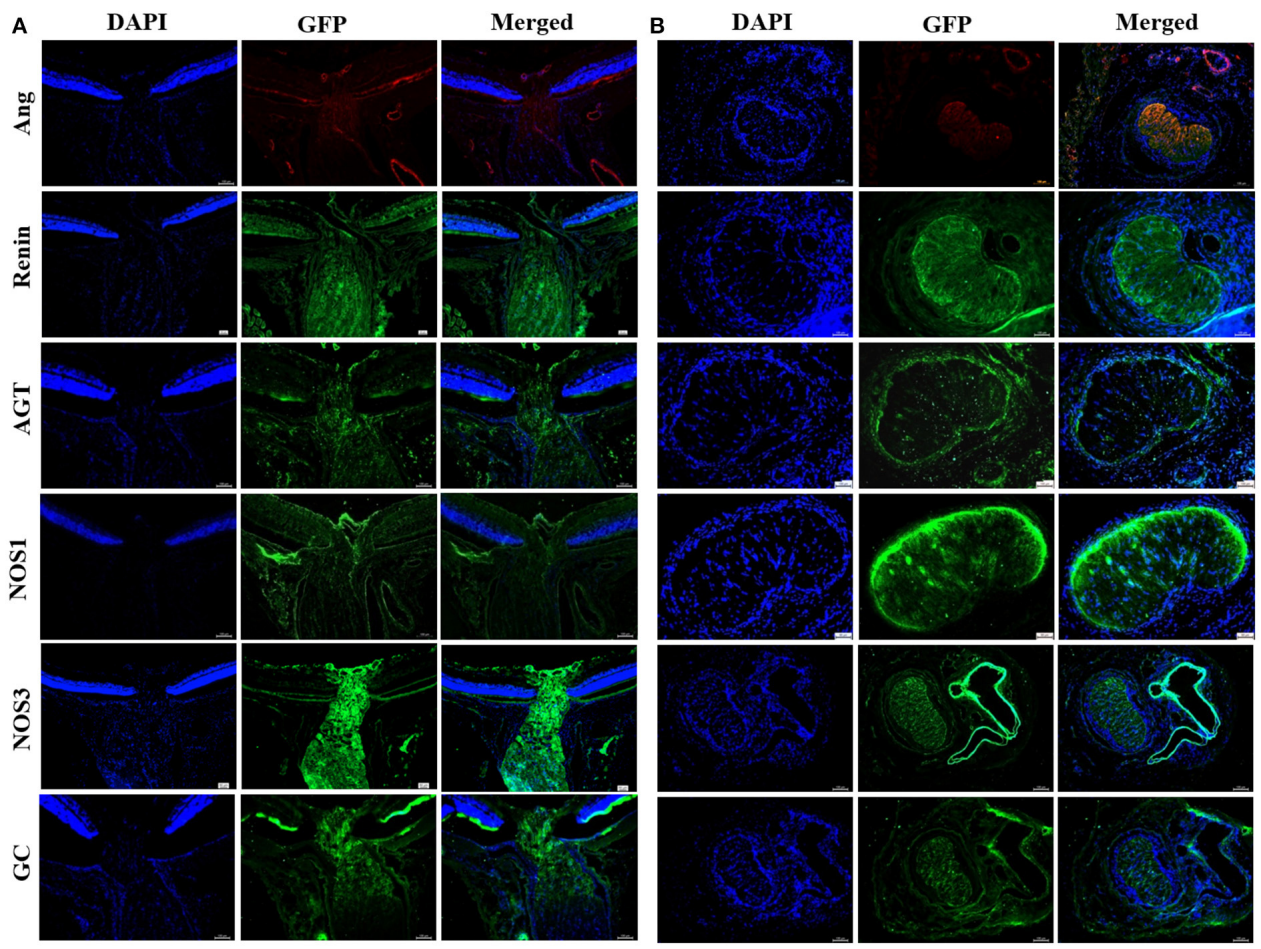
Immunofluorescent staining of NOS and RAS components in rat LC tissues. **(A)** Longitudinal section of LC tissue showed positive staining of Ang, renin, AGT, NOS1, NOS3, and GC. **(B)** Horizontal section of LC tissue showed positive staining of Ang, renin, AGT, NOS1, NOS3, and GC. LC, Lamina cribrosa; Ang, Angiotensin; AGT, Angiotensinogen; NOS, Nitric oxide synthase; GC, Guanylate cyclase.

The histological characteristics of rats' LC are presented in Figure 1. Representative immunofluorescent staining for vasoactive substances LC tissues are illustrated in Figures 2, 3. Immunofluorescence double staining against GFAP and endothelin-1 (ET-1) demonstrated obvious consistencies in tissue distribution within the whole optic nerve (Figure 2). Figure 3 depicts the immunofluorescence detection of nitric oxide synthase (NOS) and renin-angiotensin system (RAS) components both in the longitudinal and horizontal sections of LC tissue.

#### Hyperoxia induced vasoconstriction in rat retina

**Figure 4:**
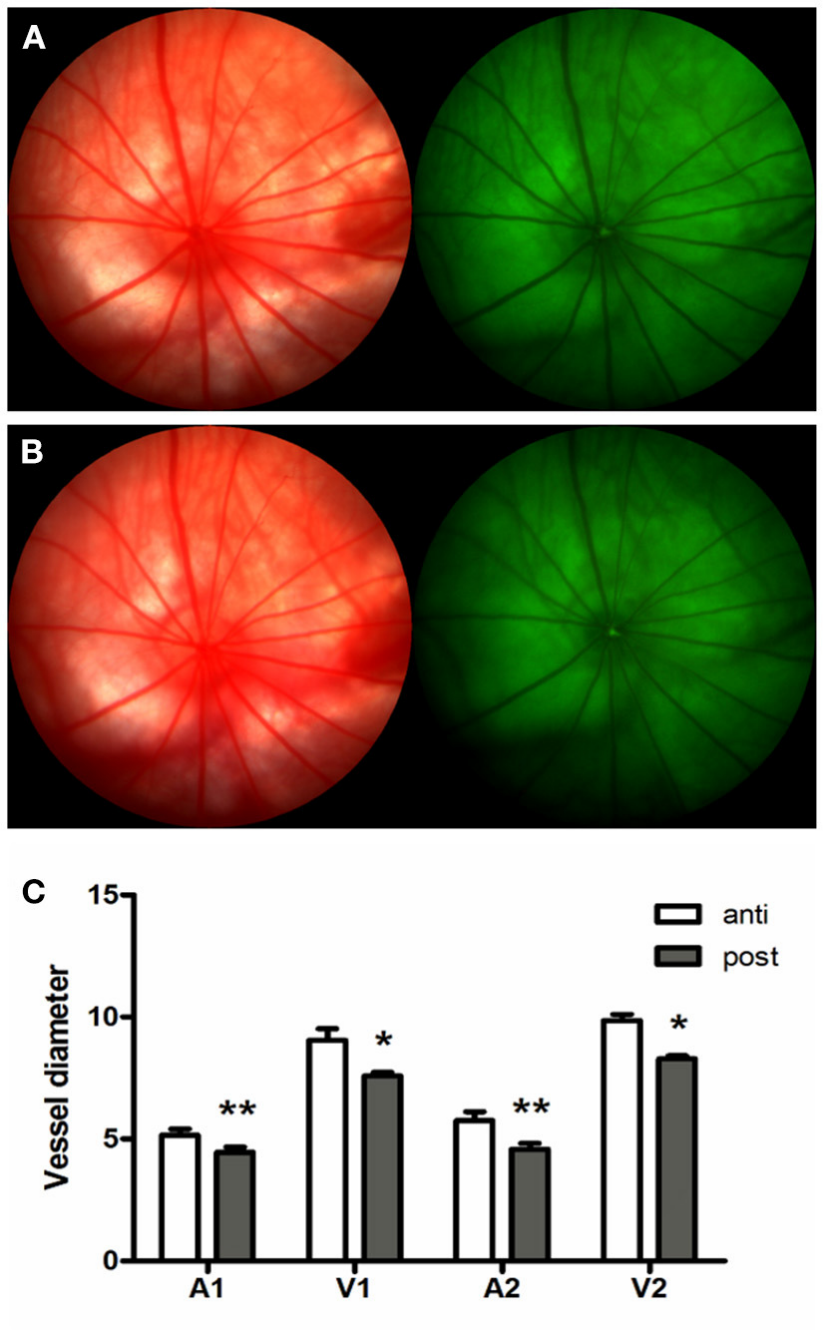
Hyperoxia induced vasoconstriction in rat retina. **(A)** Fundus imaging and FFA of rat retina proir to experimental processing. **(B)** Fundus imaging and FFA of rat retina after consecutive hyperoxia exposure for 7 days. **(C)** Variation of retinal vessel diameter induced by hyperoxia exposure. *p < 0.05, **p < 0.01, FFA, Fundus fluorescein angiography; A1 and V1, Arterioles and venules beside the ONH edge; A2 and V2, Arterioles and venules at one time the optic disc diameter.

To investigate the vascular response in hyperoxia, adult rats were exposed to consecutive hyperoxia for 7 days. Before hyperoxia exposure, the diameter of arterioles (A1) and venules (V1) beside the ONH edge was 5.16 ± 0.50 pixels, and 9.05 ± 0.95 pixels, arterioles (A2), and venules (V2) at one time the optic disc diameter was 5.76 ± 0.72 and 9.85 ± 0.51 pixels, respectively. The corresponding values after hyperoxia exposure for 7 days were then shown to be 4.44 ± 0.44, 7.58 ± 0.32, 4.59 ± 0.47, and 8.30 ± 0.23 pixels. Retinal vessels demonstrated significant contraction after being exposed to consecutive hyperoxia (Figure 4).

#### Hyperoxia inhibited NO production in tissues

In normoxia, the NO level in the retina and LC were fund to be 65.32 ± 4.04 and 29.87 ± 2.58 μM/L. The corresponding values in rats exposed to consecutive hyperoxia for 7 d were then shown to be 53.05 ± 4.97 and 24.02 ± 2.38 μM/L, respectively, with a significant difference between the two groups (*p* < 0.05). Accordingly, hyperoxia obviously was found to inhibit NO production in tissues.

#### Hyperoxia induced vasoconstriction in rat retina *via* up-regulation of ET-1 and RAS and down-regulation of NOS

**Figure 5:**
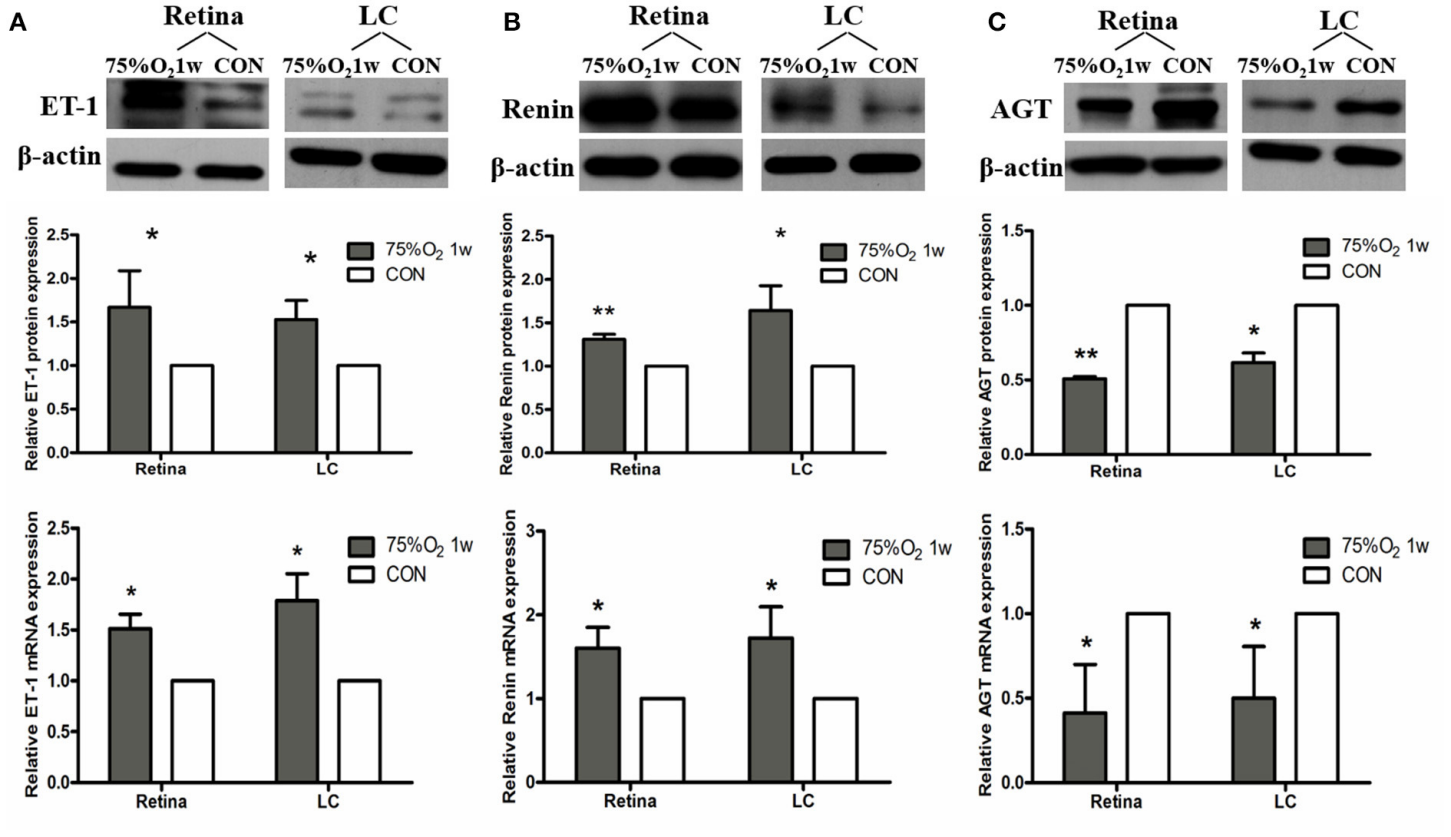
Hyperoxia induced vasoconstriction in rat retina *via* up-regulation of ET-1 and RAS. **(A)** Protein and mRNA expression of ET-1 in retina and LC significantly increased after 75%O_2_ exposure for 7 days. **(B)** Protein and mRNA expression of renin in retina and LC significantly increased after 75%O_2_ exposure for 7 days. **(C)** Protein and mRNA expression of AGT in retina and LC significantly decreased after 75%O_2_ exposure for 7 days. *p < 0.05, **p < 0.01, LC, Lamina cribrosa; ET-1, Endothelin 1; RAS, Renin-angiotensin system.

**Figure 6:**
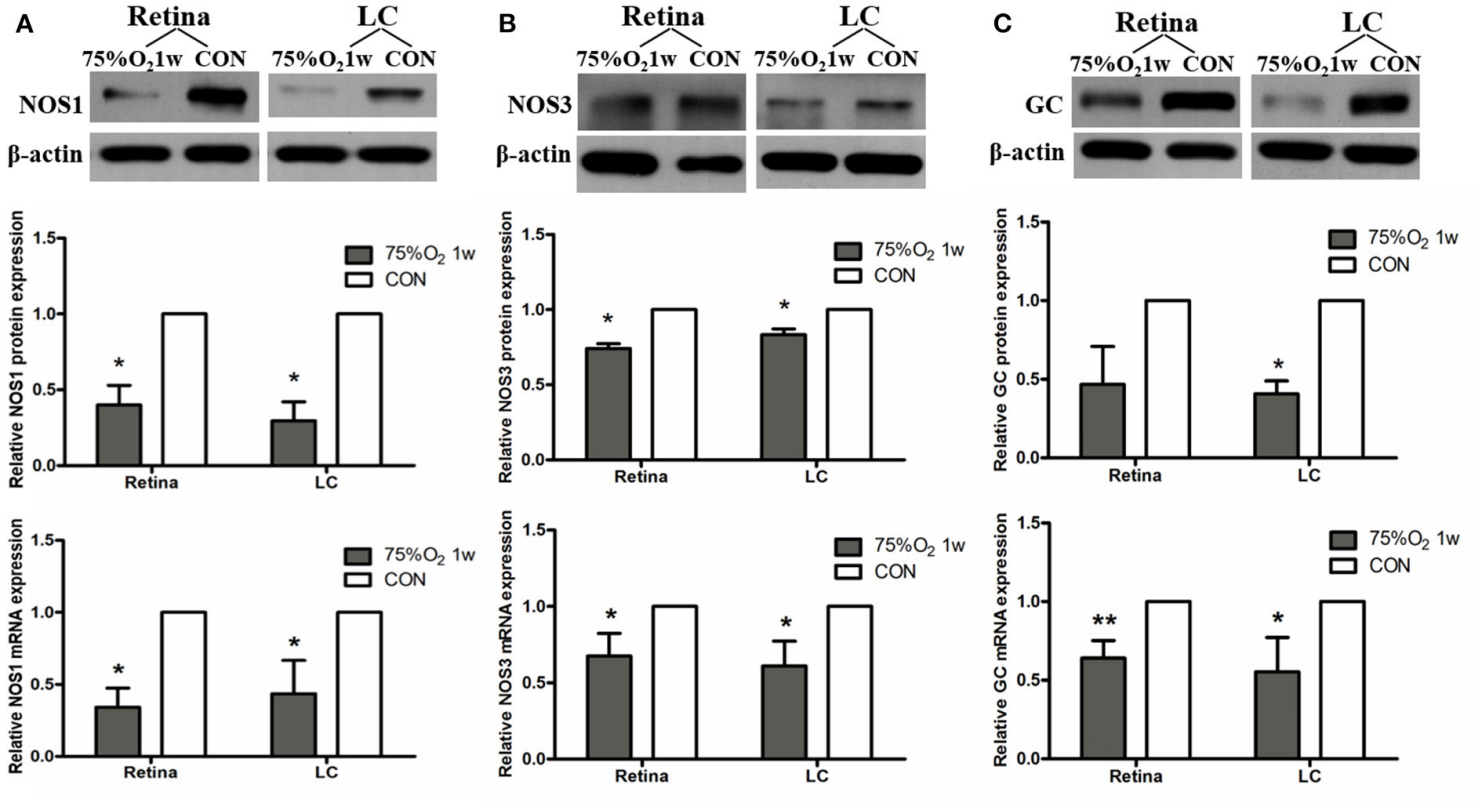
Hyperoxia induced vasoconstriction in rat retina *via* down-regulation of NOS. **(A)** Protein and mRNA expression of NOS1 in retina and LC significantly decreased after 75%O_2_ exposure for 7 days. **(B)** Protein and mRNA expression of NOS3 in retina and LC significantly decreased after 75%O_2_ exposure for 7 days. **(C)** Protein and mRNA expression of GC in retina and LC significantly decreased after 75%O_2_ exposure for 7 days. *p < 0.05, **p < 0.01, LC, Lamina cribrosa; NOS, Nitric oxide synthase; GC, Guanylate cyclase.

The expression of protein and mRNA of ET-1 in tissues was shown to be significantly increased after 75%O_2_ exposure for 7 days (*p* < 0.05). Meanwhile, the protein and mRNA expression of renin exhibited a similar trend with ET-1 (*p* < 0.05). In contrast, the protein and mRNA expression of NOS and guanylate cyclase (GC) in the hyperoxia group were found to be down-regulated compared with that of control (*p* < 0.05), as shown in Figures 5, 6.

### *In vitro* results

#### Isolation and identification of ONH astrocytes from rat LC explants

**Figure 7:**
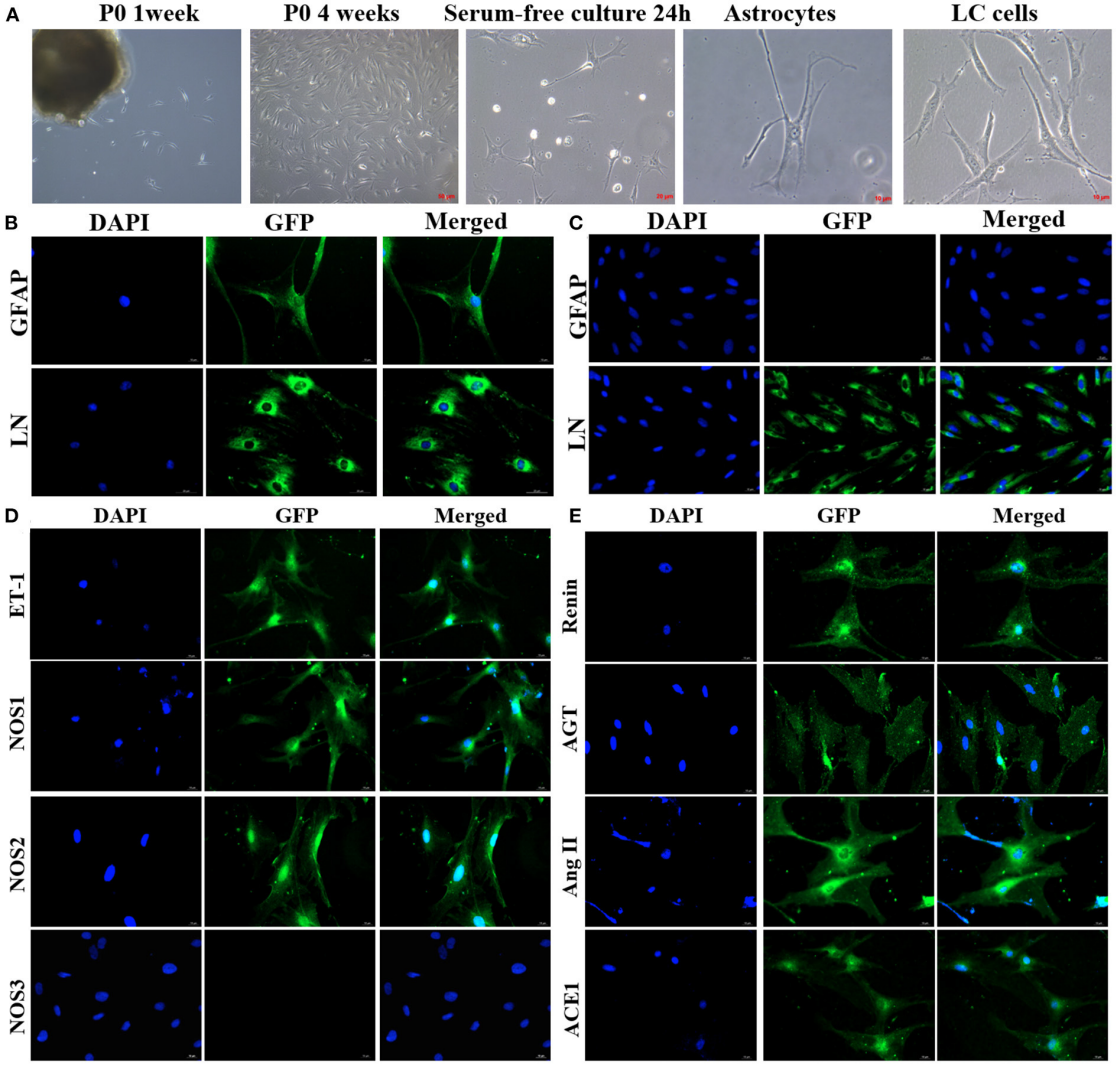
Characterization of ONH astrocytes from explants of the rat LC plants. **(A)** Cells grew out of the explants of the LC within 1 week and reached confluence at about 4 weeks. ONH astrocytes had several processes with star shape morphology. LC cells appeared as flat, broad shaped cells. **(B)** ONH astrocytes showed positive staining for GFAP and LN. **(C)** LC cells showed negative staining for GFAP and positive staining for LN. **(D,E)** ONH astrocytes exhibited positive staining for ET-1, NOS1, NOS2, rennin, AGT, Ang, and ACE1, with negative staining for NOS3. ONH, Optic nerve head; LC, Lamina cribrosa; ET-1, Endothelin 1; GFAP, Glial fibrillar acid protein; LN, Laminin; ET-1, Endothelin 1; NOS, Nitric oxide synthase; AGT, Angiotensinogen; Ang, Angiotensin; ACE1, Angiotensin converting enzyme 1.

Two distinct cell types grew out of the rat LC explants within 1 week, reaching confluence at about 4 weeks. ONH astrocytes were successfully separated and purified. ONH astrocytes grew as large stellate cells with conspicuous thin, long and varicose processes, and had a large round nucleus with one or two nucleoli. Immunofluorescent staining for GFAP was shown to be positive in this cell type. LC cells grew as polygonal, flat cells with a prominent, oval nucleus containing nucleoli, and displayed negative staining for GFAP. Both cell types were positively stained for LN (Figures 7A–C).

#### Expression of vasoactive substances in ONH astrocytes isolated from rat LC explants

Immunofluorescent staining for the vasoactive substances in ONH astrocytes is shown in Figures 7D,E. ONH astrocytes exhibited positive staining for ET-1, NOS1, and NOS2, and negative staining for NOS3. In addition, representative components of RAS were also detected. Rennin, angiotensinogen (AGT), angiotensin (Ang), and angiotensin converting enzyme-1 (ACE1) were strongly expressed both in nucleus and cytoplasm of ONH astrocytes.

#### Hyperoxia induced VSMCs contraction in a direct co-culture system

**Figure 8:**
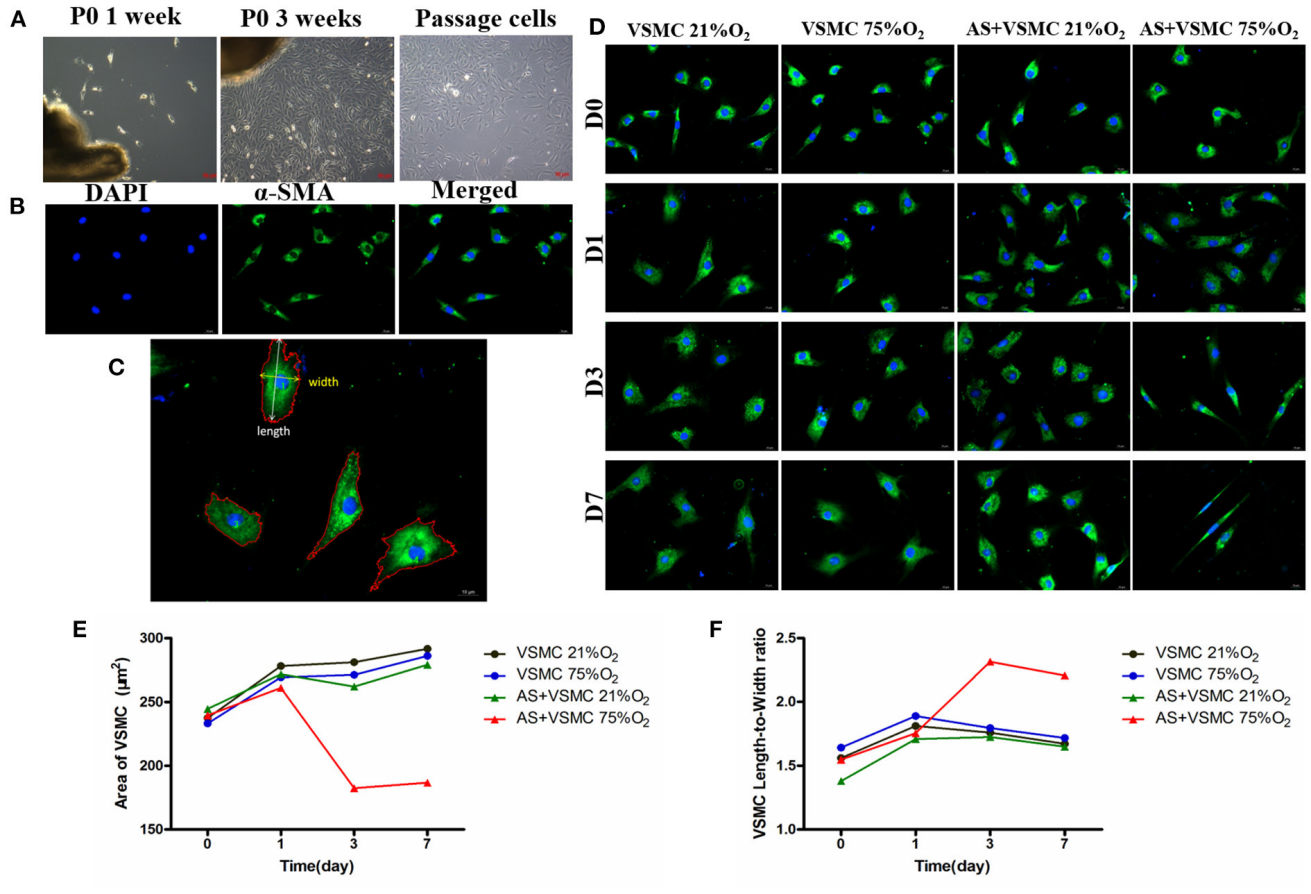
Isolation and identification of VSMCs and contraction of VSMCs in direct co-culture systems induced by hyperoxia. **(A)** Primary VSMCs grew out of the explants within 1 week, and reached confluence in about 3 weeks. Passaged VSMCs grew as a “hill and valley” pattern. **(B)** VSMCs showed positive staining for α-SMA. **(C)** The area, length and width of α-SMA-positive cells were analyzed. **(D)** Morphology of VSMCs in different culture conditions. **(E)** Area of VSMCs significantly decreased in co-culture systems after hyperoxia exposure for 3 days and 7 days. **(F)** VSMCs length-to-width ratio significantly increased in co-culture systems after hyperoxia exposure for 3 and 7 days. VSMCs, Vascular smooth muscle cells; α-SMA, alpha-smooth muscle actin.

Hyperoxia was found to induce VSMCs contraction in a co-culture system (Figure 8). The morphometric parameters of VSMCs are shown in the extended Table 1. In the ONH astrocytes and VSMCs direct co-culture system, the length-to-width ratio was observed to be significantly increased in hyperoxia at days 3 and 7, while the area of VSMCs exhibited a decreasing trend compared with that of normoxia. While cultured alone, the ratio and area of VSMCs showed no difference between hyperoxia and normoxia conditions. Results of the pairwise comparison between groups are shown in the extended Table 2.

#### Hyperoxia inhibits NO production in ONH astrocytes

**Figure 9:**
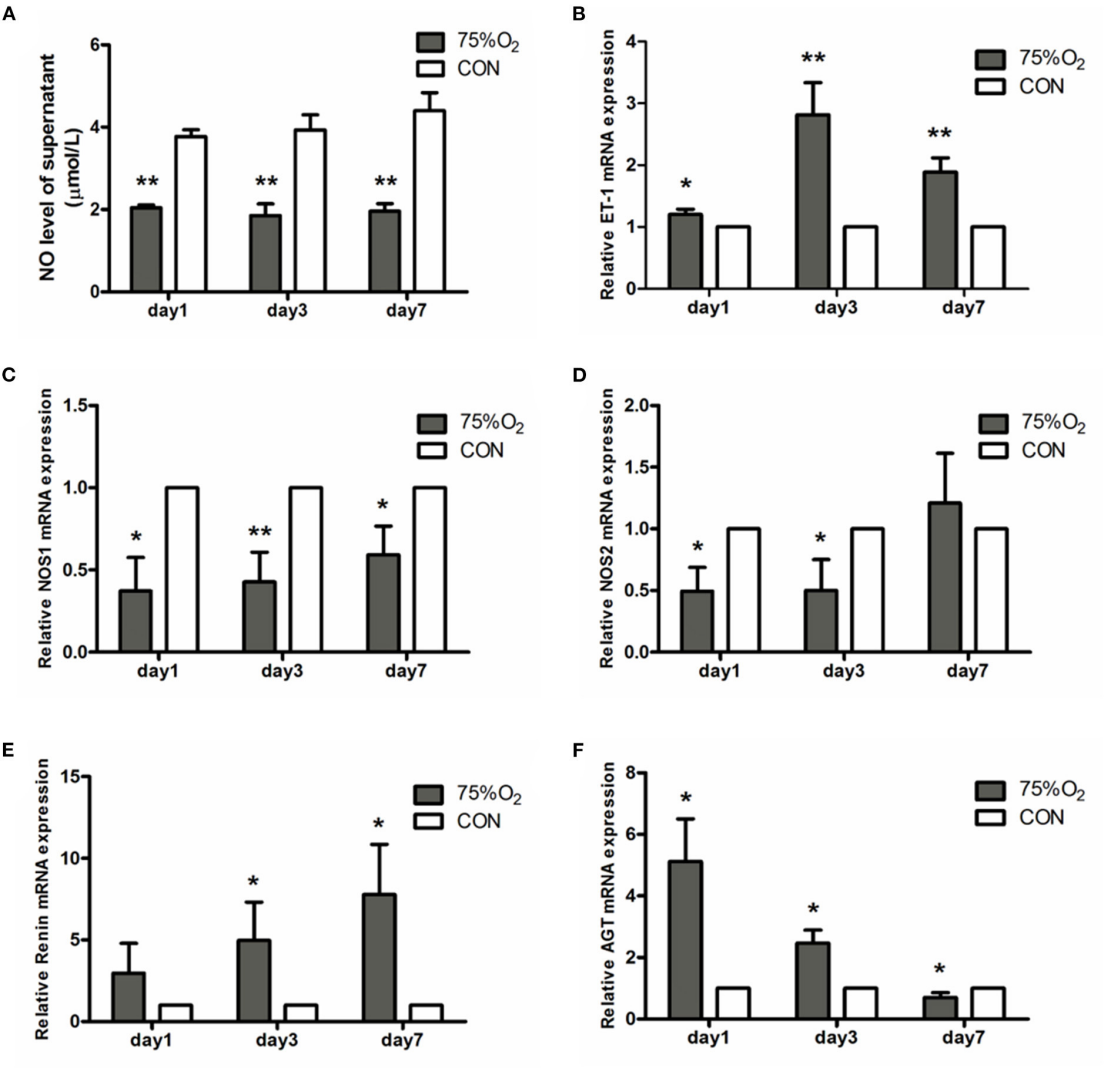
NO levels of supernatant and relative mRNA expression of vasoactive substances in ONH astrocytes after hyperoxia culturing. **(A)** Hyperoxia inhibited NO production in astrocytes. The NO level of supernatant significantly decreased in 75%O_2_ on days 1, 3, and 7. **(B)** Real-time PCR showed that the genetic expression of ET-1 was obviously increased in hyperoxia. **(C,D)** Real-time PCR demonstrated that the genetic expression of NOS was obviously decreased in hyperoxia. **(E,F)** Real-time PCR showed that the genetic expression of RAS was obviously increased in hyperoxia. *p < 0.05, **p < 0.01, NO, Nitric oxide; RAS, Renin-angiotensin system.

The changes in NO levels are shown in Figure 9A. NO levels in the supernatant of ONH astrocytes on day 1 were found to be 2.02 ± 0.18 μmol/L at 75%O_2_ and 4.22 ± 0.46 μmol/L at 21%O_2_. The corresponding values on days 3 and 7 were shown to be 1.47 ± 0.20 and 1.69 ± 0.27 μmol/L at 75%O_2_, 4.32 ± 0.91 μmol/L, and 4.5 ± 0.82 μmol/L at 21%O_2_, respectively. Accordingly, NO levels significantly decreased in hyperoxia on days 3 and 7 (*p* < 0.05).

#### Gene Expression of ONH astrocytes in hyperoxia

Representative factors associated with vascular regulation were selected for real-time PCR (Figure 9). ET-1 (Edn1) showed an obvious rising trend in hyperoxic stress (*p* < 0.05). In contrast, NOS1 (Nos1) was found to be decreased in hyperoxia than in normoxia. NOS2 (Nos2) showed a decrease in culture condition 75%O_2_ on days 1 and 3 (*p* < 0.05), though it increased on day 7. Ren (Renin) exbihibited a consistent rising trend in hyperoxia than that of the control. Meanwhile, angiotensinogen (Agt) was shown to increase in culture condition of 75%O_2_ on days 1 and 3 (*p* < 0.05), though it decreased on day 7 (*p* < 0.05).

## Discussion

The present study decribed the abundance in expression of vasoactive substances within rat LC tissue and ONH astrocytes. According to the vivo study, rat retinal vessels were showed to have significant contraction following exposure to consecutive hyperoxia. Meanwhile, *in vitro*, hyperoxia was found to induce VSMCs contraction in the ONH astrocytes and VSMCs direct co-culture system. Moreover, ET-1, NO and components of RAS may serve as the potential factors involved in both vasoconstriction *in vivo* and VSMCs contraction *in vitro*. The corresponding findings of this study suggested that LC, especially ONH astrocytes, play an important role in vascular associated effects.

The histological results of this study showed that the structure of the rat ONH was lamellar, which had well-structured collagen bundles in the longitudinal section and a typical mesh-like structure in the transverse section. The cross section of the CRV mostly formed a “D” or crescentic shape adjacent to the artery in the LC region, equivalent to that of humans (18). Kang et al. (19) decribed an arterial-like appearance of the venous endothelium in the posterior LC and suggested that changes implicate the posterior LC as a site of altered hemodynamic stress. In addition, the immunofluorescence assay demonstrated obvious positive staining of ET-1, NOS, rennin, and Ang, with all factors being present in have been found in ocular tissues (20–23). The special structural features and abundant expression of vacoactive substances provide advantages for the LC to participate in ocular blood flow regulation.

Astrocytes are the predominant glia cell type in the non-myelinated ONH and have significant uniformity with respect to astrocyte and capillary constitution (24). Accordingly, this may implicate astrocytes' importance in governing the structural characteristics of ONH. The quantitative properties of astrocytes in the LC region of rats shared similarities to the human eye. Additionally, the microvasculature of the rat ONH has several similarities with that of the primate (25). The rat ONH provides distinct advantages in studying human optic nerve diseases. Hernandez et al. (15) first carried out cell cultures from human LC and described the morphological characteristics of two cell types isolated from LC. Lambert (4) then improved the method of culturing, and the two distinct cell types were successfully separated by a serum free culture. Drawing on previous methods, ONH astrocytes were successfully isolated from rat LC in the study. Moreover, cell identification was performed with immunofluorescent staining for GFAP and LN (8, 24). As a result, this method may be a reliable technique that can be used to better understand the potential function of ONH astrocytes.

This study utilized immunofluorescent staining in order to detect the expression of vascular tone regulation associated factors in LC tissue and ONH astrocytes. According to the obtained results, both LC tissue and primary cultured ONH astrocytes were found to express ET-1, NOS, and RAS components. ET-1 is a potent vasoactive peptide that has been demonstrated to be present and functionally active in the eye (20). ET-1 affects both ophthalmic vessels and smaller vessels due to their effects on smooth muscle cells and pericytes (26). In addition, ET-1 contributes to the vasoconstriction of human retinal arterioles under both normoxic and hyperoxic conditions (27). The corresponding results showed that staining of ET-1 was positive in primary ONH astrocytes. Additionally, double staining for GFAP and ET-1 exhibited obvious consistencies in tissue distribution in rat LC tissues. ET-1 mRNA detected in LC tissue and primary ONH astrocytes suggested that astrocytes may secrete ET-1. Haefliger (27) described the expression of mRNA by ONH astrocytes for ETA and ETB receptors as well as preproET-1, suggesting that these cells may serve as a source for ET-1 in the ONH.

NO and ET-1 play major roles in the control of ocular blood flow (28, 29). Nitric oxide synthase inhibition has been shown to reduce basal blood flow in the ONH, which indicates that endogenous NO is involved in the regulation of basal vascular tone in the ONH (30). This study showed that LC tissue and ONH astrocytes expressed abundant levels of NOS. NO levels in the tissue and supernatant of ONH astrocytes were found to be significantly decreased in hyperoxia. Previous studies have also found that NO contributes to retinal vascular regulation in hypoxia and hyperoxia (31, 32). In addition, increased retinal blood flow in response to flicker stimulation may be mediated by NO production *via* NOS1 activation (33).

GC-1, the downstream target of NO, has been known to be widely expressed in the retina of multiple species (34). GC was detected in both the transverse and longitudinal sections of the whole optic nerve in this study, thus confirming the important role of the NO-GC pathway in ONH physiology.

The RAS is well-known for its role in the regulation of blood pressure as well as in fluid and electrolyte homeostasis. Various histopathological and molecular biological evidences have shown the presence of RAS autocrine mechanism in the eye (35). The Mas receptor protein is specifically expressed in retinal ganglion cells, nerve fibers, Müller cells, and endothelial cells (36). However, it remains unclear whether renin is expressed in ocular astrocytes. Accordingly, the results of this study showed that primary ONH astrocytes and LC tissue expressed renin, AGT, angiotensin and ACE1. Moreover, the expression of renin was observed to be upregulated in hyperoxia both *in vitro* and *in vivo*. In contrast, protein and mRNA expression of AGT in the retina, LC tissue and ONH astrocytes were found to be significantly decreased after 75%O_2_ exposure for 7 days. This finding may be related to the massive amount of renin convert from prorenin. Therefore, these results provide direct evidence at the cellular level for the existence of RAS in ONH. Furthermore, individual components of the RAS may act as potential therapeutic targets for the treatment of ocular disease such as glaucoma (37, 38).

Astrocytes are the main glial cell type in ONH, with their end feet forming a layer around the vascular wall. Though the contractile response of VSMCs in the co-culture system was shown to provide direct evidence for the involvement of ONH astrocytes in vascular regulation. The main limitation of this study is the lack of relevant experiments for functional verification. In our future study, loss-of-function and gain-of-function experiments will be conducted to provide more evidence to support the involvement of ONH in vascular regulation.

This is the first study to report the expression of various vasoactive substances by ONH astrocytes and LC tissue. And the study preliminarily explored the possible involvement of ONH astrocytes in vascular associated effects. The precise role and mechanism of ONH astrocytes in vascular associated effects is complex. However, this study provides a novel direction for future studies.

## Data availability statement

The original contributions presented in the study are included in the article/supplementary material, further inquiries can be directed to the corresponding author.

## Ethics statement

The study was approved by the animal experimental Ethics Committee of Zhongshan Ophthalmic Center, Sun Yat-sen University.

## Author contributions

YD designed the study, interpreted data, and wrote the manuscript. QG designed the study, interpreted data, and revised the manuscript. YD, YF, XQ, LL, YY, YL, and WS performed the experiments. YD, XQ, YF, and LL contributed to data analysis. All authors contributed to the article and approved the submitted version.

## Funding

This study was supported by the National Science and Technology Pillar Program of the Twelfth Five-year Plan (2012BAI08B00) and the National Natural Science Foundation of China (81271008).

## Conflict of interest

The authors declare that the research was conducted in the absence of any commercial or financial relationships that could be construed as a potential conflict of interest.

## Publisher's note

All claims expressed in this article are solely those of the authors and do not necessarily represent those of their affiliated organizations, or those of the publisher, the editors and the reviewers. Any product that may be evaluated in this article, or claim that may be made by its manufacturer, is not guaranteed or endorsed by the publisher.

## References

[1] Tovar-Vidales T Wordinger RJ Clark AF. Identification and localization of lamina cribrosa cells in the human optic nerve head. Exp Eye Res. (2016) 147:94–7. 10.1016/j.exer.2016.05.006 27167365

[2] Lopez NN Clark AF Tovar-Vidales T. Isolation and characterization of human optic nerve head astrocytes and lamina cribrosa cells. Exp Eye Res. (2020) 197:108103. 10.1016/j.exer.2020.108103 32522476 PMC7483633

[3] Liu X Clark AF Wordinger RJ. Expression of ciliary neurotrophic factor (CNTF) and its tripartite receptor complex by cells of the human optic nerve head. Mol Vis. (2007) 13:758–63. 17563726 PMC2768760

[4] Lambert W Agarwal R Howe W Clark AF Wordinger RJ. Neurotrophin and neurotrophin receptor expression by cells of the human lamina cribrosa. Invest Ophthalmol Vis Sci. (2001) 42:2315–23. 11527945

[5] Lambert WS Clark AF Wordinger RJ. Neurotrophin and Trk expression by cells of the human lamina cribrosa following oxygen-glucose deprivation. BMC Neurosci. (2004) 5:51. 10.1186/1471-2202-5-51 15579199 PMC539236

[6] Li H Bui BV Cull G Wang F Wang L. Glial cell contribution to basal vessel diameter and Pressure-Initiated vascular responses in rat retina. Invest Ophthalmol Vis Sci. (2017) 58:1–8. 10.1167/iovs.16-20804 28055098 PMC5225997

[7] Shibata M Sugiyama T Kurimoto T Oku H Okuno T Kobayashi T. Involvement of glial cells in the autoregulation of optic nerve head blood flow in rabbits. Invest Ophthalmol Vis Sci. (2012) 53:3726–32. 10.1167/iovs.11-9316 22589427

[8] Attwell D Buchan AM Charpak S Lauritzen M Macvicar BA Newman EA. Glial and neuronal control of brain blood flow. Nature. (2010) 468:232–43. 10.1038/nature09613 21068832 PMC3206737

[9] Marina N Christie IN Korsak A Doronin M Brazhe A Hosford PS. Astrocytes monitor cerebral perfusion and control systemic circulation to maintain brain blood flow. Nat Commun. (2020) 11:131. 10.1038/s41467-019-13956-y 31919423 PMC6952443

[10] Paisley CE Kay JN. Seeing stars: Development and function of retinal astrocytes. Dev Biol. (2021) 478:144–54. 10.1016/j.ydbio.2021.07.007 34260962 PMC8542354

[11] O'Sullivan ML Punal VM Kerstein PC Brzezinski JT Glaser T Wright KM. Astrocytes follow ganglion cell axons to establish an angiogenic template during retinal development. Glia. (2017) 65:1697–716. 10.1002/glia.23189 28722174 PMC5561467

[12] Zahs KR Wu T. Confocal microscopic study of glial-vascular relationships in the retinas of pigmented rats. J Comp Neurol. (2001) 429:253–69.3. 10.1002/1096-9861(20000108)429:2<253::AID-CNE6>3.0.CO;2-S 11116218

[13] Garcia-Bermudez MY Freude KK Mouhammad ZA van Wijngaarden P Martin KK Kolko M. Glial cells in glaucoma: friends, foes, and potential therapeutic targets. Front Neurol. (2021) 12:624983. 10.3389/fneur.2021.624983 33796062 PMC8007906

[14] Shinozaki Y Koizumi S. Potential roles of astrocytes and muller cells in the pathogenesis of glaucoma. J Pharmacol Sci. (2021) 145:262–7. 10.1016/j.jphs.2020.12.009 33602506

[15] Hernandez MR Igoe F Neufeld AH. Cell culture of the human lamina cribrosa. Invest Ophthalmol Vis Sci. (1988) 29:78–89. 3275593

[16] Kaja S Payne AJ Naumchuk Y Levy D Zaidi DH Altman AM. Plate reader-based cell viability assays for glioprotection using primary rat optic nerve head astrocytes. Exp Eye Res. (2015) 138:159–66. 10.1016/j.exer.2015.05.023 26048476 PMC4553084

[17] Leik CE Willey A Graham MF Walsh SW. Isolation and culture of arterial smooth muscle cells from human placenta. Hypertension. (2004) 43:837–40. 10.1161/01.HYP.0000119191.33112.9c 14967841

[18] Taylor AW Sehu W Williamson TH Lee WR. Morphometric assessment of the central retinal artery and vein in the optic nerve head. Can J Ophthalmol. (1993) 28:320–4. 8313218

[19] Kang MH Balaratnasingam C Yu PK Morgan WH McAllister IL Cringle SJ. Morphometric characteristics of central retinal artery and vein endothelium in the normal human optic nerve head. Invest Ophthalmol Vis Sci. (2011) 52:1359–67. 10.1167/iovs.10-6366 21071729

[20] Salvatore S Vingolo EM. Endothelin-1 role in human eye: a review. J Ophthalmol. (2010) 2010:354645. 10.1155/2010/354645 21461356 PMC3065050

[21] Laspas P Goloborodko E Sniatecki JJ Kordasz ML Manicam C Wojnowski L. Role of nitric oxide synthase isoforms for ophthalmic artery reactivity in mice. Exp Eye Res. (2014) 127:1–8. 10.1016/j.exer.2014.06.018 25017185

[22] Holappa M Vapaatalo H Vaajanen A. Local ocular renin-angiotensin-aldosterone system: Any connection with intraocular pressure? A comprehensive review. Ann Med. (2020) 52:191–206. 10.1080/07853890.2020.1758341 32308046 PMC7877937

[23] Holappa M Vapaatalo H Vaajanen A. Many faces of renin-angiotensin system - focus on eye. Open Ophthalmol J. (2017) 11:122–42. 10.2174/1874364101711010122 28761566 PMC5510558

[24] Balaratnasingam C Kang MH Yu P Chan G Morgan WH Cringle SJ. Comparative quantitative study of astrocytes and capillary distribution in optic nerve laminar regions. Exp Eye Res. (2014) 121:11–22. 10.1016/j.exer.2014.02.008 24560677

[25] Morrison JC Johnson EC Cepurna WO Funk RH. Microvasculature of the rat optic nerve head. Invest Ophthalmol Vis Sci. (1999) 40:1702–9. 10393039

[26] Haefliger IO Meyer P Flammer J Luscher TF. The vascular endothelium as a regulator of the ocular circulation: a new concept in ophthalmology? Surv Ophthalmol. (1994) 39:123–32. 10.1016/0039-6257(94)90157-0 7801220

[27] Haefliger IO Flammer J Beny JL Luscher TF. Endothelium-dependent vasoactive modulation in the ophthalmic circulation. Prog Retin Eye Res. (2001) 20:209–25. 10.1016/S1350-9462(00)0002-3 11173252

[28] Hayreh SS. Blood flow in the optic nerve head and factors that may influence it. Prog Retin Eye Res. (2001) 20:595–624. 10.1016/S1350-9462(01)00005-2 11470452

[29] Schmetterer L Polak K. Role of nitric oxide in the control of ocular blood flow. Prog Retin Eye Res. (2001) 20:823–47. 10.1016/S1350-9462(01)00014-3 11587919

[30] Schmidl D Boltz A Kaya S Lasta M Pemp B Fuchsjager-Mayrl G. Role of nitric oxide in optic nerve head blood flow regulation during isometric exercise in healthy humans. Invest Ophthalmol Vis Sci. (2013) 54:1964–70. 10.1167/iovs.12-11406 23439596

[31] Kringelholt S Holmgaard K Bek T. Relaxation of porcine retinal arterioles during acute hypoxia *in vitro* depends on prostaglandin and NO synthesis in the perivascular retina. Curr Eye Res. (2013) 38:965–71. 10.3109/02713683.2013.794247 23768139

[32] Izumi N Nagaoka T Sato E Sogawa K Kagokawa H Takahashi A. Role of nitric oxide in regulation of retinal blood flow in response to hyperoxia in cats. Invest Ophthalmol Vis Sci. (2008) 49:4595–603. 10.1167/iovs.07-1667 18552394

[33] Yoshioka T Nagaoka T Song Y Yokota H Tani T Yoshida A. Role of neuronal nitric oxide synthase in regulating retinal blood flow during flicker-induced hyperemia in cats. Invest Ophthalmol Vis Sci. (2015) 56:3113–20. 10.1167/iovs.14-15854 25783603

[34] Wareham LK Buys ES Sappington RM. The nitric oxide-guanylate cyclase pathway and glaucoma. Nitric Oxide. (2018) 77:75–87. 10.1016/j.niox.2018.04.010 29723581 PMC6424573

[35] White AJ Cheruvu SC Sarris M Liyanage SS Lumbers E Chui J. Expression of classical components of the renin-angiotensin system in the human eye. J Renin Angiotensin Aldosterone Syst. (2015) 16:59–66. 10.1177/1470320314549791 25287897

[36] Prasad T Verma A Li Q. Expression and cellular localization of the Mas receptor in the adult and developing mouse retina. Mol Vis. (2014) 20:1443–55. 25352750 PMC4203581

[37] Wada Y Higashide T Sakaguchi K Nagata A Hirooka K Sugiyama K. Compromised blood flow in the optic nerve head after systemic administration of aldosterone in rats: a possible rat model of retinal ganglion cell loss. Curr Eye Res. (2022) 47:777–85. 10.1080/02713683.2022.2029907 35179420

[38] Hirooka K Kiuchi Y. The retinal renin-angiotensin-aldosterone system: implications for glaucoma. Antioxidants. (2022) 11:610. 10.3390/antiox11040610 35453295 PMC9029628

