# Supplementary material for: Optic nerve head astrocytes contribute to vascular associated effects
Source: Front Med (Lausanne). 2022 Jul 26;9:943986. doi: 10.3389/fmed.2022.943986 (PMC9362728; doi:10.3389/fmed.2022.943986)
Supplement: Supplementary file 1 [file Table_1.pdf]

Supplementary Material

Supplementary Table 1. Morphometric parameters of VSMCs in different culture condition.

		VSMCs 21% O_2	VSMCs 75% O_2	AS+VSMCs 21% O_2	AS+VSMCs 75% O_2
Area (μm^2)	day0	237.32±37.29	233.08±45.16	244.50±66.77	239.36±78.04
	day1	278.19±42.37	269.22±99.14	271.73±61.19	260.96±67.99
	day3	281.18±48.02	271.17±59.24	261.94±49.63	182.38±51.50
	day7	296.09±53.54	291.67±50.10	279.19±57.71	186.75±72.80
Length (μm)	day0	23.68±4.50	27.73±10.55	23.74±4.23	26.86±7.83
	day1	28.31±6.34	28.48±5.64	27.64±4.27	27.20±5.99
	day3	30.00±3.17	30.04±3.84	31.36±6.59	23.35±5.25
	day7	31.21±5.86	30.21±6.21	31.69±8.02	22.62±4.56
Width (μm)	day0	15.47±3.35	16.78±4.11	17.67±3.29	17.40±3.84
	day1	15.97±2.40	15.49±3.27	16.30±2.22	15.44±2.24
	day3	17.74±3.28	16.92±2.35	17.90±4.22	10.44±3.14
	day7	18.24±3.32	18.62±4.85	18.95±3.70	10.36±2.14
Ratio (length -to- width)	day0	1.56±0.30	1.64±0.31	1.38±0.36	1.55±0.33
	day1	1.81±0.48	1.89±0.44	1.71±0.24	1.75±0.39
	day3	1.76±0.33	1.80±0.28	1.72±0.31	2.31±0.47
	day7	1.67±0.24	1.72±0.18	1.65±0.18	2.21±0.37

Supplementary Table 2. P values of comparison between co-culture&hyperoxia and other groups.

		AS+VSMCs 75%O_2 **vs**		
		VSMCs 21%O_2	VSMCs 75%O_2	AS+VSMCs 21%O_2
Area	day0	1	1	1
	day1	0.98	1	0.999
	day3	*0.002*	*0.013*	*0.014*
	day7	*0.008*	*0.01*	*0.033*
Length	day0	0.833	1	0.836
	day1	0.995	0.988	1
	day3	*0.021*	*0.027*	*0.044*
	day7	*0.011*	*0.036*	*0.041*
Width	day0	0.786	0.999	1
	day1	0.995	1	0.939
	day3	*0*	*0*	*0.002*
	day7	*0*	*0.002*	*0*
Ratio (length -to- width)	day0	1	0.984	0.853
	day1	1	0.973	1
	day3	*0.042*	*0.049*	*0.02*
	day7	*0.009*	*0.013*	*0.005*
